# Changes in inflammatory biomarkers in the nasal mucosal secretion after septoplasty

**DOI:** 10.1038/s41598-022-20480-5

**Published:** 2022-09-28

**Authors:** Marn Joon Park, Yong Ju Jang

**Affiliations:** Department of Otorhinolaryngology - Head and Neck Surgery, Asan Medical Center, University of Ulsan College of Medicine, 388-1 Pungnap 2-dong, Songpa-gu, Seoul, 138-736 Korea

**Keywords:** Anatomy, Biomarkers

## Abstract

Deviated nasal septum (DNS) is suggested to be associated with nonspecific inflammation of the nasal mucosa. The authors hypothesized septoplasty may reduce nasal mucosal inflammation, therefore the authors aimed to measure various inflammatory biomarkers in the nasal secretion following septoplasty. Prospectively, 17 patients undergoing elective septoplasty were included. Symptomatic changes after septoplasty were evaluated with Sino-nasal Outcome Test (SNOT-22) and Nasal obstruction symptom evaluation (NOSE) scores. Using acoustic rhinometry, changes of the nasal airway volume were measured. Nasal secretion was collected within 2 weeks and 3 months before and after septoplasty, respectively. The inflammatory biomarker high-mobility group box 1 (HMGB1) and vasoactive intestinal peptide (VIP), and inflammatory cytokines including tumor necrosis factor α (TNF α), interferon γ (IFN-γ), interleukin-4 (IL-4), eotaxin-1, and regulated upon activation, normal T cell expressed and presumably secreted (RANTES) were quantified in the nasal secretion by enzyme-linked immunosorbent assays or multiplex bead array assays. The patients' mean age was 30.5 ± 6.8 (ranging from 19 to 43), consisting of 15 male and 2 female patients. The median SNOT-22 and NOSE scores changed from 54 to 14 and 78 to 15, respectively, both showing a significant decrease. In acoustic rhinometry, nasal cavity volume of convex side significantly increased after septoplasty, whereas significant discrepancy of nasal airway volume between concave and convex sides became insignificant. No significant difference was noted both before and after septoplasty between the concave and convex sides in all seven biomarkers. The HMGB1, RANTES, IL-4, and TNF-α concentrations following septoplasty showed significant decrease in 34 nasal cavities of 17 patients (all p < 0.05). However, when the 17 concave and 17 convex sides were analyzed separately, the significant reduction in four biomarkers were only significant in the concave sides (all p < 0.05), but not significantly reduced in convex sides. Septoplasty may have benefited not only in normalizing the nasal airflow and symptom improvement, but also in nonspecific inflammation attenuation in the nasal airway.

## Introduction

Deviated nasal septum (DNS) is responsible for nasal obstruction (NO) symptoms^1^. NO is not only related to mechanical alteration in the nasal airflow but is also associated with nasal mucosa inflammation^2^. Despite the high DNS incidence and its frequent encounter in ENT practice, the effects of DNS on the nasal mucosa are not yet fully understood.

Only a few studies discussing the pathological condition of nasal mucosa in DNS patients have been currently noted. Jang et al.^3^ reported increased lymphocytic infiltration and decreased mucociliary clearance and secretory cells on the concave side of the nasal mucosa compared with the convex side. Kamani et al.^4,5^ reported a significantly higher portion of squamous metaplasia in the concave side mucosa than the normal group who did not have DNS. Polat et al.^6^ and Ulusoy et al.^7^ reported the recovery of nasal mucociliary function after septoplasty.

Additionally, previous studies have stated that DNS is a risk factor for chronic rhinosinusitis (CRS) development^8^. In allergic rhinitis (AR) patients with DNS, septoplasty resulted in significant relief in allergic symptoms (i.e., rhinorrhea, sneezing, and itching)^9,10^. These previous findings suggest that DNS patients may not only have mechanically increased nasal airway resistance but also nonspecific nasal mucosa inflammation.

The clinical benefits of the straightened nasal septum with the equalized nasal volume of both nasal cavities had been reported by numerous publications for many decades with various septoplasty techniques^1,11–14^. Dinis et al.^11^ and Giles et al.^12^ reported nasal obstruction improvement as measured by a subjective questionnaire after septoplasty. Uslu et al.^15^ argued the beneficial aspects of septoplasty by showing the improvement of the ciliary function of the nasal mucosa after septoplasty at 3 months. These findings may suggest a decrease in nasal mucosa inflammation after septoplasty.

The presence of inflammation in a certain human tissue may be represented by changes in some biomarkers (e.g., cytokines or chemokines). However, no prior studies have discussed the changes in the nasal mucosal inflammation in terms of changes in the biomarkers in the nasal mucosal secretion after septoplasty. Therefore, the authors aimed to reveal the inflammatory cytokine profiles in patients with DNS and find out whether septoplasty could improve the inflammatory condition of the nasal mucosa.

## Materials and methods

### Subjects

From 2017 January to 2017 February, patients between 16 and 60 years old, diagnosed with DNS with symptomatic NO in the outpatient clinic, and scheduled for an elective septoplasty were prospectively recruited. The diagnosis of DNS was made by examination using the anterior rhinoscopy with endoscopic evaluation. Patients with a history of tobacco smoking, evidence of combined sinonasal disorders other than DNS shown on the preoperative computed tomography scan (i.e., chronic sinusitis and nasal polyposis), previous sinonasal surgery, trauma, chronic systemic disease (i.e., malignant neoplasm, diabetes, bronchial asthma, autoimmune disease, and chronic hepatitis), and patients administrating any medications consecutively over 3 months before surgery (i.e., antibiotics, nonsteroidal anti-inflammatory drugs, glucocorticoids, and antidepressants) were excluded from the study.

Among 24 patients who agreed to participate in the study, four patients withdrew from the study after septoplasty. Moreover, three patients had submucosal radiofrequency ablation of the inferior turbinate during septoplasty based on the surgeon’s clinical judgment. Thus, the three patients were withdrawn from the study because the ablated turbinate mucosa may alter the study results. Patients who develop complications in the postoperative period following septoplasty, or patients with acute upper respiratory infection (URI) upon preoperative or 3-months postoperative final visit upon collection of the nasal secretion were planned to exclude from the final analysis.

A total of 17 patients were enrolled in the final analysis. All study participants were given informed consent. No patients complained of URI symptoms in the preoperative or 3-months follow-up visit. The mean age ± standardized deviation was 30.5 ± 6.84 years old (range, 19–43 years old). Of the patients, 15 (88%) were males and two (12%) were females, respectively. All patients were tested with a skin prick test for 12 common allergens to determine the presence of AR. The diagnosis of AR was confirmed in 10 (56%) patients.

The study was approved by the Asan Medical Center institutional review board (AMC-IRB) (Investigation No. S2017-0309–0002). With a strict monitoring of the AMC-IRB, this study was delivered in accordance with the relevant guidelines and regulatory protocols of AMC-IRB.

### Surgical procedure

In all subjects, the septoplasty was performed by a single surgeon (Y. J. Jang) using the methods elaborated in the previously published paper^13^. Briefly, under general anesthesia, a hemitransfixion incision was made on the concave side mucosa. Following the mucoperichondrial flap elevation, deviated quadrangular cartilage was partially resected with preservation of the L-strut, and deviated bony septum (vomer, maxillary crest, and/or perpendicular plate of the ethmoid bone) were resected. Bony batten graft was designed with the harvested bone and sutured on the caudal septum of the concave side followed by the closure of the mucoperichondrial flap. All patients simultaneously underwent out-fracture lateralization of both inferior turbinates. All patients were routinely administered with 1 g ampicillin/0.5 g sulbactam intravenous antibiotics for prophylactic antibiotics, 325 mg acetaminophen every 8 h with 500 mg amoxicillin and potassium clavulanate equivalent to 125 mg of clavulanic acid every 8 h per oral, from the postoperative day 1–5.

### Outcome measurements

The subjective assessment of septoplasty outcomes was evaluated by applying the validated, patient-reported outcome measurements: the SinoNasal Outcome Test (SNOT-22)^16^ for the overall nasal symptom assessment and Nasal Obstruction Symptom Evaluation (NOSE) scores^17^ were adopted to assess the symptoms especially related with nasal obstruction. Preoperative measurements were made within 2 weeks before surgery, and postoperative measurements were 3 months after septoplasty.

**Figure 1:**
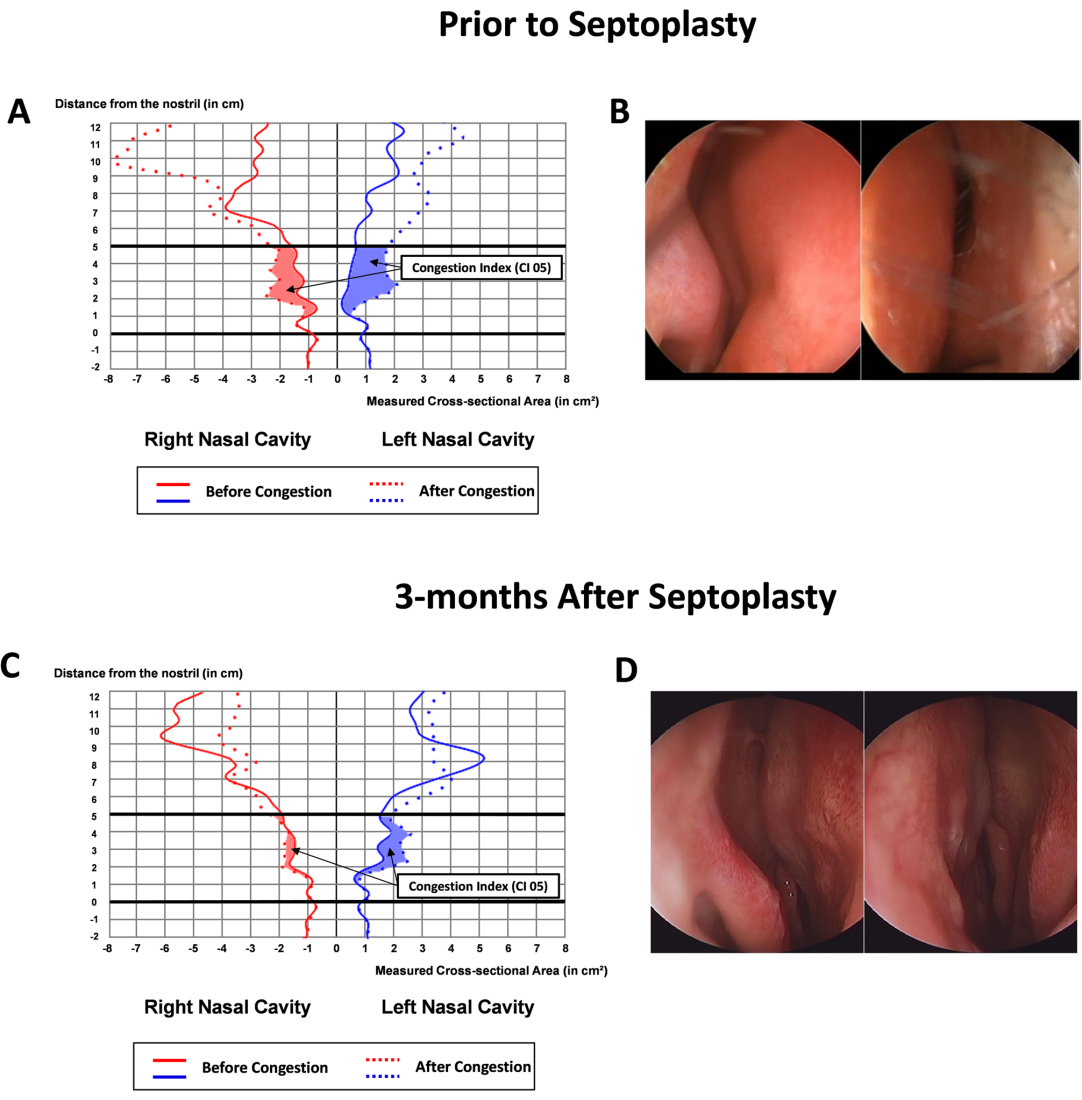
Evaluation of the nasal airway after septoplasty. An endoscopic examination with the acoustic rhinometry evaluation in a 27-year old male patient with the deviated nasal septum. Visualization of the narrowed nasal airway on the left side is well observed with the endoscopic exam (**B**) and acoustic rhinometry (**A**). The nasal septum is well straightened with the widening of the left nasal cavity 3 months after septoplasty with bilateral lateralization of the inferior turbinate (**D**). Equalization of the bilateral nasal airway was achieved as shown on the acoustic rhinometry (**C**). Remarkably, the swelling of the nasal mucosa has decreased (**B**, **D**) as shown in the decrease in the congestion index on the acoustic rhinometry (**A**, **C**).

Evaluation of the changes in the nasal geometry after septoplasty was done by acoustic rhinometry (GM Instruments Ltd, Kilwinning, UK). Patients were asked to sit, the nosepiece connected to the external adapter was placed against the nares, and soundproofing lubricants were applied to seal the nostril without nose distortion. The volumetric measurements (in cubic centimeter) of the nasal cavity were recorded on each side one at a time, both before and after applying the commercialized decongestant (xylometazoline nasal spray; Otrivin 1 mg/mL, Novartis, Basel, Switzerland). The nasal cavity volume of 0–5 cm from the nostril (NCV05) was measured (Fig. 1). As suggested in the study by Kjaergaard et al.^18^, a congestion index of 0–5 cm from the nostril (CI05) was used in the current study for the quantification of the degree of nasal mucosal congestion in AR as follows: [(decongested NCV05 − baseline NCV05)/baseline NCV05] × 100 (%).

### Nasal secretion collection and inflammatory cytokine quantification

**Figure 2:**
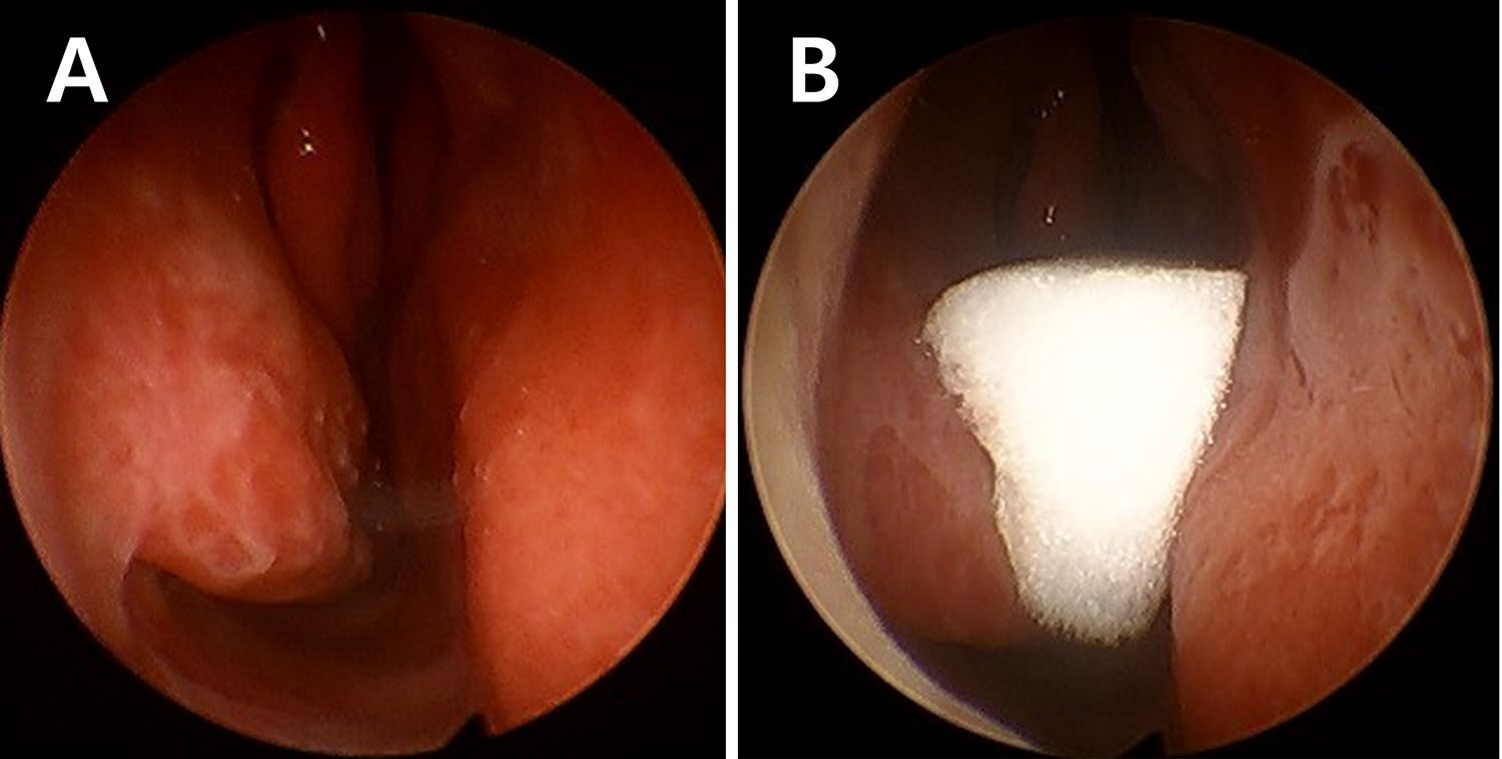
Nasal secretion collection. Upon visualization of the nasal cavity with the 30-degree endoscope, 20 mm × 10 mm × 5 mm cubic piece of synthetic polyurethane sponge (NasoPore) cubic piece was placed in the space between inferior turbinate, middle meatus, and nasal septum. The inserted cube was removed after 10 min in each nasal cavity.

Nasal secretion was collected in each nasal cavity at the outpatient setting applying the following instruction similar to the method validated in the previous literatures^19,20^. The authors used a commercialized absorbable polyurethane sponge, NasoPore (Polyganics, Rozenburglaan, the Netherlands), to collect the nasal secretion. NasoPore was fabricated into 20 mm × 10 mm × 5 mm cubic pieces using sterilized scissors. Before inserting the designed NasoPore piece, patients were asked to blow their nose to remove secretion. Using the sterilized bayonet forceps, the fabricated NasoPore piece was placed in the space between the inferior turbinate, nasal septum, and anterior border of the middle turbinate, securing the air passage for patients’ nasal breathing (Fig. 2). The fabricated NasoPore piece was carefully inserted with a guidance of an anterior rhinoscope and rigid nasal endoscope to minimize the nasal mucosal irritation upon insertion, and there was no patient who sneezed upon insertion of the NasoPore piece. Each piece was inserted in both nasal cavities simultaneously, and the NasoPore pieces were extracted after 10 min. The removed NasoPore pieces were immediately fully submerged in a 20-mL room-temperature 0.9% saline solution for 10 min. After removal of the cubic pieces in the solution, the solution was centrifuged at 1000 rpm (157×*g*) for 10 min. Aliquot supernatant was immediately stored at − 70 °C.

All samples were analyzed with enzyme-linked immunosorbent assay (ELISA) for the quantification of the high-mobility group box 1 protein (HMGB1) and human vasointeractive peptide (VIP). Multiplex bead array assays (MBAA) was applied to measure the concentrations of interleukin-4 (IL-4), tumor necrosis factor-alpha (TNF-α), interferon-gamma (IFN-γ), Eotaxin-1/(c–c motif chemokine ligand (CCL) 11, and regulated on activation normal T-cell expressed and secreted (RANTES)/CCL 5 in the nasal secretion. In addition, 100 µL of an aliquot in duplicates was used for the quantification of each cytokine, following the manufacturers' instructions (HMGB1 ELISA kit, Chondrex, Inc., WA, USA; VIP ELISA kit, Phoenix Pharmaceuticals, Inc., Burlingame, CA, USA; MBAA, Human Luminex assay kit, R&D Systems, Minneapolis, MN, USA). The obtained results were evaluated with Bio-Plex Manager MP/Bio-Plex Manager 6.1 software (Bio-Rad Laboratories, Inc., Hercules, CA, USA). The cutoff value range for each biomarker are as follows: HMGB1 (0.01–0.99 ng/mL); VIP (0.15–2.32 ng/mL); IL-4 (13.59–3825.77 pg/mL); TNF-α (8.86–2161.11 pg/mL); IFN-γ (48.32–11,998.86 pg/mL); Eotaxin-1/CCL11 (60.61–14,787.62 pg/mL); and RANTES/CCL5 (20.56–5052.09 pg/mL).

### Statistical analysis

To determine the significant difference in NCV05 and CI05 between the concave and the convex sides, the concave side NCV05 and CI05 were paired with the convex side. A Wilcoxon signed-rank test was used to observe the significant differences between the convex and concave sides. In addition, the pre- and postoperative NCV05 and CI05 were paired, and a Wilcoxon signed-rank test was used to observe the significant differences between the pre- and postoperative NCV05 and CI05.

The SNOT-22 and the NOSE scores in the preoperative and 3-months postoperative period were paired, and a Wilcoxon signed-rank test was used to observe the significant differences after septoplasty.

To evaluate the statistically significant differences in the biomarker concentration in the nasal secretion between the paired concave and convex sides, a Wilcoxon signed-rank test was adopted to compare each biomarker concentration between the 17 paired nasal cavities. The comparison was made in the preoperative samples as well as in the 3-months postoperative samples. In addition, the significance in the difference of each biomarker concentration between the AR group and patients without the AR group was measured using the Mann–Whitney test.

To determine the statistically significant changes in each biomarker concentrations after septoplasty, the preoperative and the postoperative biomarker concentrations in each nasal cavities in 17 patients were paired, making a total number of 34 nasal cavities paired pre- and post- operatively. Depending on the normal distribution of each biomarker concentration determined by D'Agostino-Pearson omnibus (k^2^) test, a paired t-test or Wilcoxon signed-rank test was applied to determine the statistical significance in each biomarker concentration after septoplasty.

All of the values were represented as median (interquartile range). All of the statistical tests were executed in a two-tailed manner. A *p* value < 0.05 was accepted as statistically significant. All the statistical analyses were performed using GraphPad Prism (version 9.2.0; GraphPad Software, San Diego, CA, USA).

## Results

### Subjective outcomes of septoplasty

In all 17 subjects, significant decrease in both SNOT-22 and NOSE scores were noted after surgery (Table 1). The median (interquartile range) of SNOT-22 score changed from 54.0 (35.5–61.5) to 14.0 (6.0–30.5), (*p* < 0.001), whereas the changes in NOSE score was from 77.5 (61.3–85.0) to 15.0 (5.0–25.0), (*p* < 0.001). The significant decrease in both SNOT-22 and NOSE scores following septoplasty indicate significantly improved nasal symptoms following septoplasty.

**Table 1:** Patient-related outcome measurements (PROM) following septoplasty.

N = 17	Prior to septoplatsy	3-months after septoplasty	*P* value*
SNOT-22 scores (ranging from 0 to 110)	54.0 (35.5–61.5)	14.0 (6.0–30.5)	** < 0.001**
NOSE scores (ranging from 0 to 100)	77.5 (61.3–85.0)	15.0 (5.0–25.0)	** < 0.001**

Significant values are in bold.

All values are shown in median, (interquartile range).

*P value calculated by using the Wilcoxon signed rank test between the pre-operative values and post-operative scores.

*NOSE* Nasal obstruction symptom evaluation, *SNOT* Sino-nasal Outcome Test.

### Acoustic rhinometry; measurement of nasal cavity volume and congestion index

The median NCV05 of the concave and the convex sides was significantly different (concave side, 5.67 mL; convex side, 4.05 mL; *p* = 0.009) before septoplasty, whereas the significant difference was not observed after surgery (concave side, 6.8 mL; convex side, 6.23 mL, *p* = 0.268; Table 2). The NCV05 of the convex side significantly increased after septoplasty (preoperative, 4.05 mL; postoperative, 6.23 mL; *p* = 0.001). Therefore, the wideness of the convex side and evenness of both nasal cavity volumes after septoplasty was verified as shown in Fig. 1.

**Table 2:** Acoustic rhinometry measurements: changes in the intranasal volume, and congestion index following septoplasty.

N = 17	Prior to septoplatsy	3-months after septoplasty	*P* value^†^
**Baseline NCV05 (ml)**			
Concave side	5.67 (4.54–6.41)	6.85 (4.76–7.63)	0.094
Convex side	4.05 (3.08–5.29)	6.23 (4.42–7.01)	**0.001**
*P* value*	**0.009**	0.268	
**CI 05 (%)**			
Concave side	48.89 (37.56–93.84)	27.52 (10.78–66.11)	**0.017**
Convex side	72.42 (44.10–117.30)	24.40 (10.12–59.45)	**0.013**
*P* value*	0.241	0.952	

Significant values are in bold.

*NCV 05* Nasal Cavity Volume of 0 to 5 cm from the nostril, *CI 05* congestion index of 0 to 5 cm from the nostril, calculated as following; [(NCV 05 After Decongestion − Baseline NCV 05)/Baseline NCV 05] × 100 (%).

All values are shown in median (interquartile range).

*P value calculated by using the Wilcoxon signed rank test between the convex side and the concave side of the nasal cavity.

^†^P value calculated by using the Wilcoxon signed rank test between the pre-operative and post-operative acoustic rhinometry measurements.

The median CI05 value did not show significant difference between the concave and convex sides both preoperatively (concave side, 48.89; convex side, 72.42; *p* = 0.241) and postoperatively (concave side, 27.52; convex side, 24.40, *p* = 0.952; Table 2). However, a significant reduction in the congestion index after septoplasty were observed in both concave (48.89%–27.52%; *p* = 0.017) and convex (72.42%–24.40%; *p* = 0.013) sides.

### Inflammatory cytokines in the nasal secretion

**Figure 3:**
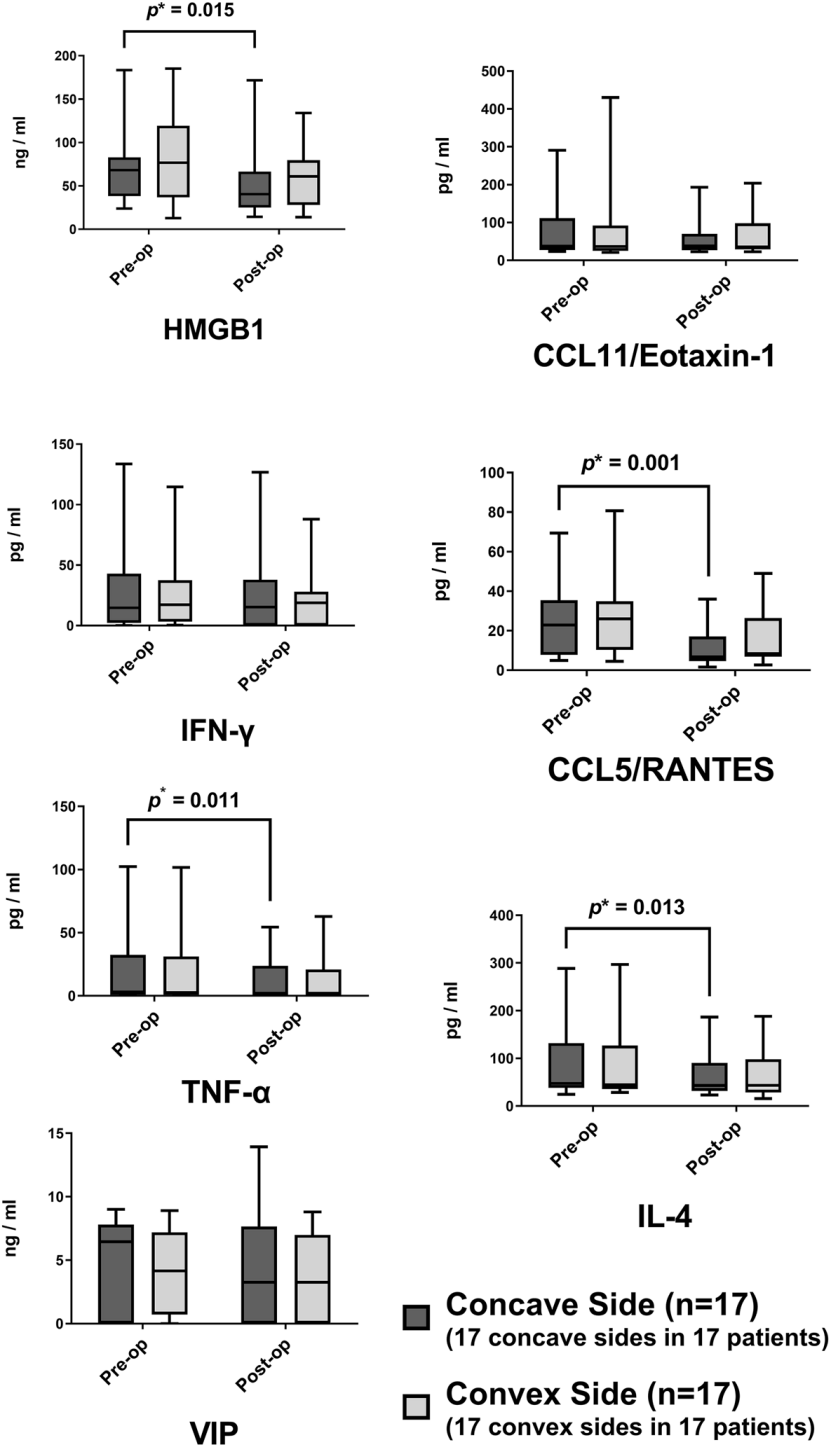
Changes in inflammatory biomarker concentrations in the nasal secretion on the concave and convex sides after septoplasty (*n* = 17). Each *box* indicates the interquartile range; the *whiskers* indicate the minimum to the maximum value. A Wilcoxon signed-rank test was used to evaluate the significant differences in each cytokine concentration between paired concave and convex nasal cavities. Similarly, the concentration of seven biomarkers in the nasal secretion before and 3 months after septoplasty were paired for all seven biomarkers, and a Wilcoxon signed-rank test was used to evaluate the significant differences between the pre- and postoperative state. *Upper asterisk* indicates statistical significance (*p* value < 0.05).

In all 17 patients, the concentrations of all seven biomarkers between the concave and the convex sides showed no significant difference (all *p* > 0.05; Fig. 3). The HMGB1, RANTES, and TNF-α concentrations showed a significant decrease after septoplasty at 3 months in the concave sides (*p* value all < 0.05), whereas the convex sides did not show significant changes in all seven biomarkers (Fig. 3).

**Figure 4:**
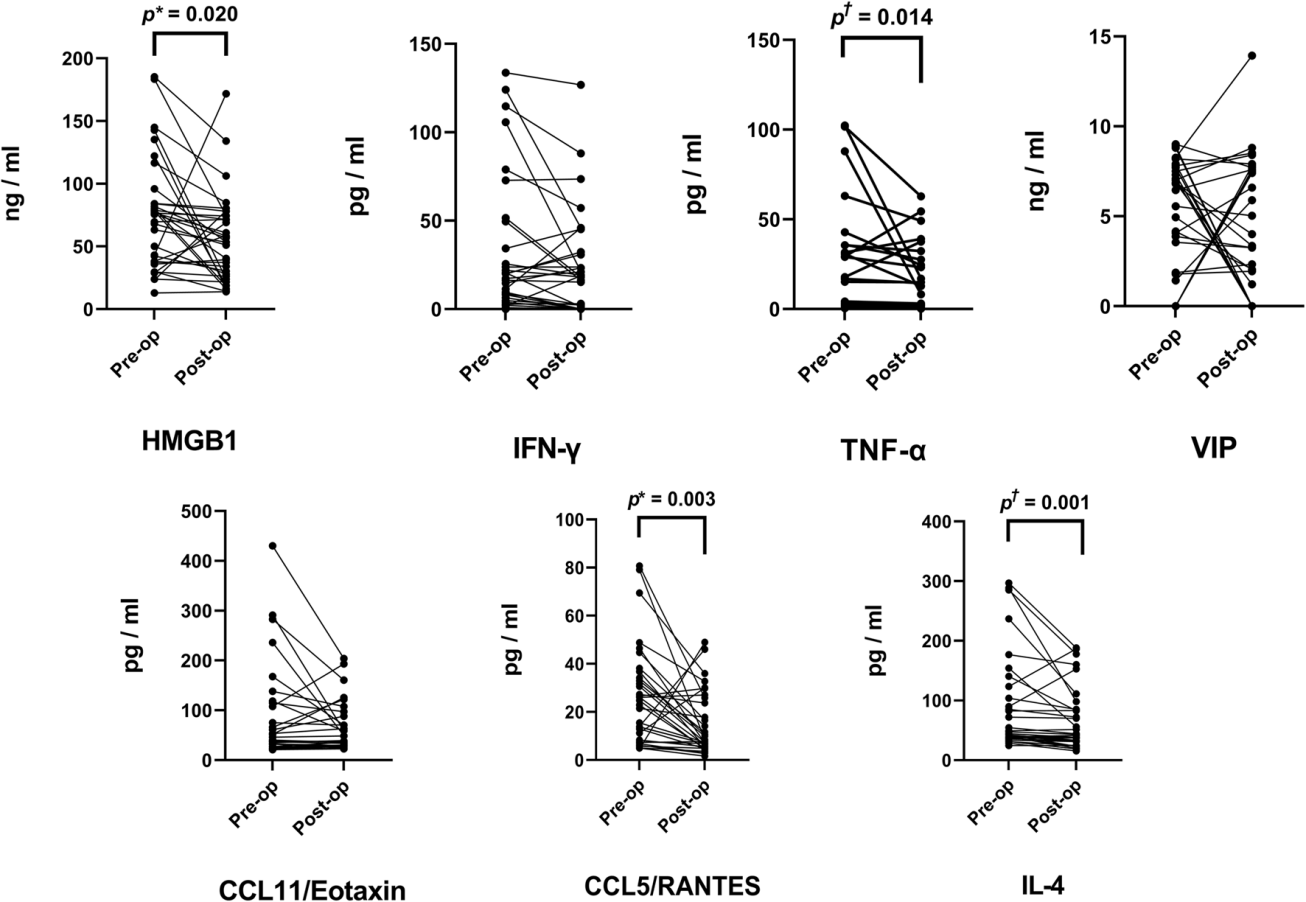
Changes in inflammatory biomarker concentration in the nasal secretion after septoplasty. Changes of each biomarker concentration in the nasal secretion after septoplasty in 34 paired nasal cavities are demonstrated. Each *dot* represents the measured biomarker concentration in each nasal cavity. A paired *t*-test or Wilcoxon signed-rank test was used to evaluate the significant differences in each cytokine concentration between the pre- and postoperative state depending on the normal distribution of the measured biomarker concentration. The *upper asterisk* indicates statistical significance by using the paired t-test (*p* value < 0.05). The *upper dagger* indicates statistical significance by using the Wilcoxon signed-rank test (*p* value < 0.05).

The concentration changes of seven inflammatory biomarkers after septoplasty of 34 nasal cavities in 17 patients are shown in Fig. 4. In the paired t-test of 34 nasal cavities for each seven biomarkers, the HMGB1, RANTES, IL-4, and TNF-α concentrations in the nasal secretion showed a significant decrease. The preoperative median (interquartile range) HMGB1 concentration was 76.1 (38.0–87.2) ng/mL, which significantly decreased to 53.4 (25.7–74.3) ng/mL (*p* = 0.001) postoperatively. Preoperative RANTES concentration was 23.8 (23.8–34.2) pg/mL, which significantly decreased to 7.5 (5.3–24.3) pg/mL after septoplasty (*p* < 0.001). The IL-4 concentration in the preoperative period was 46.1 (37.7–118.5) pg/mL, which significantly decreased to 42.2 (31.5–84.6) pg/mL after septoplasty (*p* = 0.001). Preoperative TNF-α concentration was 3.0 (0.8–30.9) pg/mL, showing a significant decrease of 1.9 (0.8–23.6) pg/mL in 3 months of postoperative period (*p* = 0.014). Moreover, the IFN-γ, VIP, and Eotaxin-1 levels did not change significantly after septoplasty (all *p* > 0.05).

**Figure 5:**
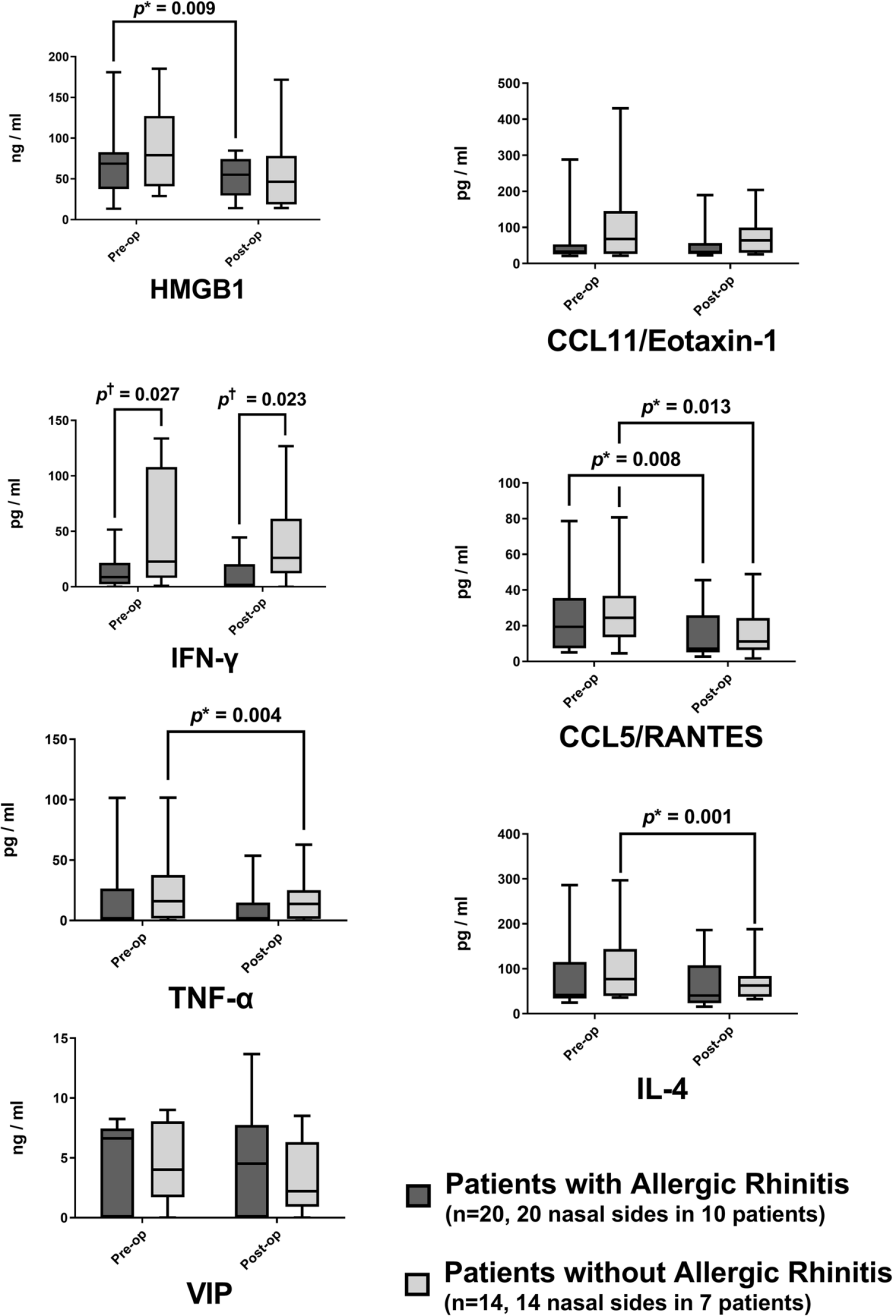
Changes in inflammatory biomarker concentration in the nasal secretion after septoplasty according to the diagnosis of allergic rhinitis (AR). Each *box* indicates the interquartile range; the *whiskers* indicate the minimum to the maximum value. Each seven pre- and the postoperative biomarker concentrations were paired, and a Wilcoxon signed-rank test was adopted to evaluate the significant changes after septoplasty in both AR and patients without AR groups. A Mann–Whitney test was applied to see the significant differences between patients with AR and without AR. The *upper asterisk* indicates statistical significance (*p* value < 0.05) calculated by the Wilcoxon signed-rank test. The *upper dagger* indicates statistical significance (*p* value < 0.05) calculated by the Mann–Whitney test.

Further breakdown of cytokine data was done to assess the effect of allergy on the results of the current study. The concentration difference of biomarkers between the patients in the AR and without AR groups showed no statistical significance (all *p* > 0.05; Fig. 5) except for the IFN-γ. The IFN-γ concentration showed significantly lower concentrations in patients in the AR than in the without AR groups in both before and after septoplasty (*p* = 0.027 and *p* = 0.023, respectively). A significant decrease in HMGB1 was observed only in the AR group (*p* = 0.009). The RANTES concentration showed a significant decrease in patients in both AR and without AR groups (*p* = 0.008 and 0.013, respectively). However, TNF-α and IL-4 showed a significant decrease only in the patients without the AR group (*p* = 0.004 and 0.001, respectively).

## Discussion

In the present study, the authors report a significant decrease in the number of inflammatory biomarkers in the nasal secretion in DNS patients after septoplasty. Objective outcomes of septoplasty were confirmed with acoustic rhinometry, showing significantly increased nasal cavity volume on the convex side and equalizing of the nasal cavity volume on both sides after septoplasty. Subjective effectiveness of septoplasty was verified with a statistically significant decrease in the SNOT-22 and the NOSE scores.

In the present study, verification of nasal inflammation was studied by assessing the concentration of various biomarkers in the nasal fluid. Nasal mucosa biopsy with quantification of the various inflammation response-related proteins or transcription factors would have been a more ideal, precise method to assess mucosa inflammation. However, harvesting the nasal mucosa especially on the nasal septum or the inferior turbinate bears a risk for unnecessary injury on the nasal mucosa and delayed healing. Therefore, with ethical consideration, the authors decided not to perform an unnecessary mucosal biopsy.

The nasal secretion collection is a noninvasive, atraumatic procedure with much lesser discomfort and does not require special equipment that can be easily done in an outpatient office setting. Similar to the methods used in the current study, Lü and Esch^19^ have proposed a nasal fluid collection method by positioning the synthetic polyurethane material in the nasal floor, which resulted in higher detectability and reproducibility of nasal cytokines than the nasal lavage method. In addition, Riechelmann et al.^20^ reported insertion of the 30 × 18 × 6 mm sized polyurethane foam in the anterior nasal cavity for 10 min, similar to our study. Their results showed the highest sensitivity and accuracy compared with nasal lavage, nasal spray-blow, or filter paper methods, in the detection of various nasal cytokine concentrations, and did not irritate the nasal mucosa at the same time^20^. Analyzing the concentrations of various cytokines in the nasal fluid in parallel using the validated methods in the previous literatures strongly supports the current study’s choice of nasal collection methods.

In acoustic rhinometry evaluation, a significant decrease in CI05 at 3 months postoperative period was observed on both sides. Interestingly, Kocak et al.^21^ reported significant reduction in CI of the inferior turbinate following total laryngectomy, in which the complete cessation of nasal airflow is being achieved. This previous reported finding and our results might support the fact that CI of the nasal mucosa is affected by the nasal airflow, and with the restoration of the normal airflow following septoplasty, a reduction in nasal mucosa CI may be achieved. In addition, congestion index (CI) of the nasal mucosa proved to show a significant positive correlation with subjective nasal obstruction^18^. The results of our study indicated that septoplasty may reduce the discrepancy in the nasal volume between the congested, and non-congested states. Our result strongly supports the beneficial effect of septoplasty in reducing the degree of mucosal swelling, thereby alleviating the NO symptom.

Previous publications discussing the nasal mucosal inflammation with DNS have only reported the lymphocytic infiltration of the nasal mucosa and alteration in normal mucociliary transportation. Therefore, the authors aimed to find the characteristics of the inflammation in the nasal mucosa of DNS patients with seven different biomarkers. To evaluate the nonspecific, overall inflammation in the nasal mucosa, HMGB1 was adopted because many previous studies reported HMGB1 and its correlation with inflammation of the sinonasal mucosa in chronic rhinosinusitis and AR. TNF-α was studied to evaluate the acute inflammation degree because it has a strong potential for initiating early or late phase inflammation in the nasal mucosa^22^. IFN-γ were evaluated to determine Th1 inflammation because IFN-γ levels are upregulated and highly secreted in the Th1 inflammation, whereas IFN-γ downregulation is observed in Th2 inflammatory condition (e.g., in AR)^23^. RANTES/CCL5 and Eotaxin-1/CCL11 are two C-C subfamily chemokines. Through the C-C chemokine receptors, RANTES and Eotaxin serve as a potent chemoattractant and activator of eosinophils, basophils, and Th2 lymphocytes^24,25^. By quantifying the RANTES and Eotaxin-1 concentration, the authors aimed to evaluate nasal inflammation especially mediated by eosinophils. IL-4 is a major cytokine involved in AR pathophysiology. IL-4 induces IgE secretion by B lymphocytes and amplifies the IgE-mediated immune response^26,27^. The quantification of IL-4 concentration in the nasal secretion was used to determine the presence of Th2 allergic inflammatory response. To find out the presence of neurogenic inflammation, VIP, a neuropeptide secreted in the parasympathetic nerve fibers in the human nasal mucosa were studied. VIP is known to play a role in nasal fluid secretion along with vasomotor tone regulation^26^.

Remarkably, a significant decrease in HMGB1 was shown after septoplasty. HMGB1 was originally known as an intranuclear transcription factor bound to the chromosomes, providing chromosome stabilization^28^. Recently, the role of HMGB1 in the initiation, and mediation in acute and chronic inflammatory responses in various organs have been revealed and is currently being emphasized by many researchers. Represented as the damage-associated molecular pattern (DAMP), this intra-nucleus protein is a key molecule promptly released into the extracellular space upon the damage of cells^28^. Considered as a hosts' immediate defense mechanism, HMGB1 binds to toll-like receptors and activates the inflammation cascade through the activation of nuclear factor-κB^29^. The presence of extracellular HMGB1 in the human airway has been reported to be associated with acute respiratory viral infection and bronchial asthma^30^. In the upper airway, previous reports are on the role of HMGB1 in sinonasal disorders. Bellussi et al.^31^ noted that HMGB1 plays a key role in chronic CRS development, and Min et al.^32^ reported a positive HMGB1 correlation with disease severity in CRS with nasal polyps. The release of HMGB1 is known to not take place in the apoptosis of the cells, rather it is released upon necrosis of the cells as a result of direct damage or viral infection^33^. Although the exact mechanism of extracellular leak of HMGB1 in human nasal mucosa is not yet fully understood, our results suggest the infiltration of HMGB1 might be resulted from some degree of mucosal injury associated with DNS. Decrease in HMGB1 following septoplasty as shown in the current study may indicate the beneficial effect of septoplasty in improving the nonspecific mucosal inflammation after septoplasty.

Interestingly, a significant decrease in TNF-α was also noticed. Widergren et al. have reported the induction of inflammatory response in human nasal mucosa by TNF-α in a dose-dependent manner^22^. Therefore, decreased TNF-α concentration noticed in the results of the current study may represent the attenuation of nonspecific nasal mucosal inflammation after septoplasty. The results of the current study show a significant decrease in RANTES and IL-4 concentration after septoplasty, which are related to the Th2 immune response. However, no significant change in Eotaxin-1 concentration was noted. In contrast, Th1-related cytokine IFN-γ showed no significant changes after septoplasty. The results of the current study may carefully suggest an attenuation of Th2 response without changes in Th1 inflammation after septoplasty. However, a more elaborated further study should be conducted in the future to clear out whether a definite Th2 response attenuation without Th1 response occurs following septoplasty.

In our study, statistically significant decrease in the RANTES and IL-4 following septoplasty were noticed, but not in the eotaxin concentrations. RANTES, IL-4, and eotaxin are the biomarkers associated with Th2 inflamamtion. In contrast, IFN-γ; increased in Th1 immune response, and VIP; a neuropeptide secreted by unmyelinated c fiber in the peripheral nerve distributed in the nasal mucosa showed no significant changes following septoplasty. RANTES, also known as a chemokine ligand 5 (CCL5), is known to be expressed in nasal epithelial cells and fibroblasts in human nasal mucosa^34^. Secreted upon chemical stimuli such as TNF-α, or IL-1, RANTES is responsible for non-selective recruitment of inflammatory cells through chemotaxis^35^. Influx of eosinophils, monocytes, memory T-cells and basophils mediate inflammation on the local tissues. The significant decrease of IL-4 and RANTES following septoplasty in our study may indicate the attenuation of Th2 inflammation with septoplasty regardless of the presence of AR. On the other hand, eotaxin is a strong, potent, selective chemotactic agent for the recruitment of eosinophils through CC chemokine receptor 3 (CCR3)^36^. Several articles have stated significant increase of eotaxin without nonsignificant increased production of RANTES in the tissues and secretions of nasal polyposis subjects^25^, which goes parallel with our results. Although our study may carefully suggest an attenuation of Th2 inflammation with septoplasty, Nevertheless, the authors cannot clarify the significant decrease of IL-4 and RANTES without eotaxin following septoplasty in the current study. Therefore, it would be an area of interest to examine the eosinophil count changes in the nasal mucosa, or the gene expression level changes of eotaxin and RANTES following septoplasty in the future studies.

The HMGB1, RANTES, IL-4, and TNF-α concentrations following septoplasty showed significant decrease in 34 nasal cavities of 17 patients (Fig. 4). However, when the 17 concave and 17 convex sides were analyzed separately, the significant reduction in four biomarkers were only significant in the concave sides (Fig. 3). Previous literatures have stated that the nasal mucosa of the DNS patients showed significant increase of lymphocyte infiltration and squamous metaplasia on both sides, especially showing more prominence damage in the concave side^3–5,37^. Our study results go in line with the previous literatures.

Recently, with the emergence of the computerized fluid dynamics (CFD) simulation model, the aerodynamics of the nasal airflow are being studied widely. Chen et al. reported an aerodynamic model focusing on the DNS in 2012^38^. In straight nasal septum simulation model, the air flow is in laminar flow with main flow shown on the space between inferior turbinate, middle meatus, and the nasal septum^38^. In contrast, DNS model showed alteration of the main airflow in both sides. In DNS model, the point of maximal air pressure, sheer stress, and the highest nasal flow rate were shown to be present at the nasal floor and superior dorsal area on the convex side, whereas it was shown on the caudal septal area and the space between the septum and middle turbinate on the concave side^38^. Concluded from their studies, altered nasal airflow in both nasal cavities as a result of DNS may directly irritate the nasal mucosa. Our study results support the probable presence of mechanical injury affecting both nasal cavities in DNS patients, which can be relieved by straightening the deviated nasal septum.

Although a significant decrease in several biomarkers in the nasal secretion after septoplasty was found, the current study has some limitations. The biomarker concentration sampled from DNS patients was not compared with samples from the normal, asymptomatic population not having DNS. Unlike electrolyte concentration in the serum which the normal reference concentration range are widely accepted, the biomarker concentration in the nasal secretion do not have a normal reference range, in addition to the fact that biomarker concentration in the nasal secretion varies within individuals. Since our study was not able to show whether a difference in the nasal secretion biomarker concentrations exists between septoplasty patients and asymptomatic healthy individuals, we would like to suggest a study in the future comparing the differences between two groups. On the other hand, the biomarker concentration in the nasal fluid in the current study was not normalized to the total protein amount in the nasal secretion, thereby possessing a possibility for an uneven distribution of collected samples.

In conclusion, a significant decrease in the HMGB1, IL-4, RANTES, and TNF-α were shown in the nasal secretion after septoplasty at 3 months, as well as improvement in overall nasal symptoms in 17 DNS patients. Improving the altered nasal airflow through septoplasty may bring about the mitigation of the nasal mucosal inflammation in DNS patients.

## Data Availability

The datasets used and/or analysed during the current study available from the corresponding author on reasonable request.
